# Supplementary material for: Health risk behaviours among people with severe mental ill health during the COVID-19 pandemic: Analysis of linked cohort data
Source: PLoS One. 2021 Oct 14;16(10):e0258349. doi: 10.1371/journal.pone.0258349 (PMC8516268; doi:10.1371/journal.pone.0258349)
Supplement: S1 File — (PDF) [file pone.0258349.s002.pdf]

University of York

OWLS

Optimising Wellbeing during Self-isolation

Thank you for agreeing to take part in this study.

Your responses will help us understand how people experience the current pandemic restrictions and how this affects their health and wellbeing.

1. Your Health and Wellbeing

In this section we would like to ask you about your physical health, mental health and wellbeing.

1.1 Compared with life before the beginning of the pandemic restrictions, how would you rate your health in general?

Physical Health

- ☐ better than before
- ☐ about the same
- ☐ worse than before
- ☐ not sure/don't know

Mental Health

- ☐ better than before
- ☐ about the same
- ☐ worse than before
- ☐ not sure/don't know

1.2 We would like to ask you four questions about how you are feeling.

There are no right or wrong answers. For each of these questions we would like you to give an answer on a scale of 0 to 10, where 0 is "not at all" and 10 is "completely".

	0	1	2	3	4	5	6	7	8	9	10
Overall, how satisfied are you with your life nowadays?											
Overall, to what extent do you feel that the things you do in your life are worthwhile?											
Overall, how happy did you feel yesterday?											

1.3 Overall, how anxious did you feel yesterday?
Please answer on a scale of 0 to 10, where 0 is "not at all anxious" and 10 is "completely anxious".

	0	1	2	3	4	5	6	7	8	9	10
Overall, how anxious did you feel yesterday?											

2. Service use

In this section we would like to understand if and how your ability to use health care services might have changed during the pandemic restrictions.

Please answer the following questions thinking about your use of health care services since the pandemic restrictions began. If you needed to use the same service more than once, please think about your most recent experience.

2.1 Your GP practice

2.1.1 Have you needed to use your GP practice?

- ☐ Yes
- ☐ No [go to question 2.2]

2.1.2 Were you able to get an appointment?

- ☐ Yes
- ☐ No [go to question 2.2]
- ☐ I haven't tried [go to question 2.2]

2.1.3 Was the appointment in person?

- ☐ In person
- ☐ Over the phone
- ☐ Online (e.g. videocall or facetime)

2.1.4 Did you receive the care/support you needed?

- ☐ Completely
- ☐ Partly
- ☐ No

2.2 Other general health services

2.2.1 Do you have any routine appointments for a physical health problem?

- ☐ Yes
- ☐ No [go to question 2.3]

2.2.2 Were you able to get an appointment?

- ☐ Yes
- ☐ No [go to question 2.3]

2.2.3 Was the appointment in person?

- ☐ In person
- ☐ Over the phone
- ☐ Online (e.g. videocall or facetime)

2.2.4 Did you receive the care/support you needed?

- ☐ Completely
- ☐ Partly
- ☐ No

2.3 Prescriptions and pharmacy/chemist services

2.3.1 Have you needed to get prescription medication?

- ☐ Yes
- ☐ No [go to question 2.4]

2.3.2 Were you able to use your usual pharmacy/chemist?

- ☐ Yes
- ☐ No

2.3.3 How did you collect your medication?

- ☐ I collected it myself
- ☐ I got a family member, friend/neighbour or volunteer to collect it
- ☐ I had it delivered
- ☐ I couldn't get it

2.3.4 Thinking of your answer above, is that how you normally collect your prescription medication?

- ☐ Yes
- ☐ No

2.4 Community mental health services

2.4.1 Have you needed to use community mental health services?

- ☐ Yes
- ☐ No [go to question 2.5]

2.4.2 Were you able to get support?

- ☐ Yes
- ☐ No [go to question 2.5]
- ☐ I haven't tried [go to question 2.5]

2.4.3 Was support given in person?

- ☐ In person
- ☐ Over the phone
- ☐ Online (e.g. videocall or facetime)

2.4.4 Did you receive the support you needed?

- ☐ Completely
- ☐ Partly
- ☐ No

2.5 Mental Health Crisis Services

2.5.1 Have you needed to use a mental health crisis service?

- ☐ Yes
- ☐ No [go to question 2.6]

2.5.2 Were you able to get support?

- ☐ Yes
- ☐ No [go to question 2.6]
- ☐ I haven't tried [go to question 2.6]

2.5.3 Was support given in person?

- ☐ In person
- ☐ Over the phone
- ☐ Online (e.g. videocall or facetime)

2.5.4 Did you receive the support you needed?

- ☐ Completely
- ☐ Partly
- ☐ No

2.6 Overall, how confident are you that support will be available from mental health services if you need it?

- ☐ Confident
- ☐ Not sure
- ☐ Not confident

2.7 Do you currently have support from mental health services?

- ☐ Yes [complete questions 2.8 and 2.9 and skip 2.10]
- ☐ No [go to question 2.10]

2.8 Since the pandemic restrictions began, the way some mental health services are provided has changed. If you have experienced any of the following changes, please tell us how you feel about them:

	I have not experienced this change	I like it better	It's OK - not better or worse	I don't like it
I had support on the telephone/ online instead of face to face appointments				
I had less frequent contact with mental health services				
I had more frequent contact with mental health services				
I had a more limited range of services or support				
I saw a different mental health worker to the person who would normally support me				
I went to a different place for appointments or support				

2.9 Thinking about the support you might need from mental health services in the year ahead, which of the following statements best fits for you?

I think I will need the same or less support from mental health services - my mental health has improved during the pandemic restrictions	
I don't think the support I need from mental health services will change - I've been ok during the pandemic restrictions	
I might need more support - the pandemic restrictions have been difficult for me but I'm going to see how things go	
I will need more support - during the pandemic restrictions my mental health has got worse	
I will need more support - I put off dealing with some things during the pandemic restrictions but now I want to get advice/help with them	
I didn't have enough support before the pandemic restrictions and I still need more support	

2.10 [Skip if 2.7 was a yes] **Thinking about the year ahead, do you think you will need support from mental health services?**

☐ Yes

☐ No

3. Everyday habits

In this section we would like to ask about smoking, drinking, diet, exercise and daily routines. Everyone has healthy habits and unhealthy habits and there are no right or wrong answers.

3.1 Please let us know how each of the following habits might have changed since the pandemic restrictions began. If you don't have any of these habits at all (e.g. you don't smoke), please choose the first option (I don't do that in general).

	I don't do that in general	More than usual	About the same	Less than usual
I drink alcohol...				
I take part in physical activity...				
I eat at least five portions of fruit and vegetables per day				
I vape or use e-cigarettes...				
I smoke....				

↓

On the table above, if you said you are not smoking, please go to question 3.5

3.2 How many cigarettes per day do you usually smoke? (Write a number below)

.... per day

3.3 How many cigarettes per day do you usually smoke? (Please choose an option)

☐ 10 or less
☐ 11 - 20
☐ 21 - 30
☐ 31 or more

3.4 How soon after you wake up do you smoke your first cigarette?

☐ Within 5 minutes
☐ 6 - 30 minutes
☐ More than 30 minutes

3.5 Since the pandemic restrictions began, have you been able to maintain a daily routine in your life?

☐ More than usual
☐ About the same
☐ Less than usual

4. COVID-19

In this section we would like to ask you if and how you have been personally affected by COVID-19.

4.1 Have you had COVID-19?

☐ Yes, diagnosed and recovered

☐ Yes, diagnosed and still ill

☐ Not diagnosed but suspected

☐ No, I don't think so

4.2 Have you ever been advised by a health professional that you are high risk or vulnerable and should self-isolate for 12 weeks (also called "shielding", that is, not leaving the house at all, even for shopping), to protect yourself from COVID-19?

☐ Yes

☐ No

4.3 Things might have changed for you throughout the pandemic restrictions. Could you please tell us which of these statements have applied to you during this time? Please choose all that apply.

☐ I was in full isolation, not leaving my home at all.

☐ I stayed at home, only leaving for exercise, food shopping, accessing medication or essential activity permitted by government guidelines.

☐ I stayed at home, only leaving for exercise, food shopping, or accessing medication AND work (e.g. as a key worker or volunteer) AND/OR other essential work or task.

☐ I was NOT following the stay-at-home recommendations but adhering to social distancing when in public (e.g. staying 2m away from others).

☐ I was NOT following the stay-at-home recommendations or social distancing when being out.

5. Social support

In this section we would like to ask you about the support you receive from people around you.

5.1 Who lives with you?

- ☐ I live on my own
- ☐ My partner or spouse
- ☐ Children under the age of 16
- ☐ Other family members
- ☐ Other people (e.g. flatmates/housemates) that are not family members and not in supported accommodation
- ☐ I live in supported accommodation

5.2 Thinking of the last two weeks, please choose an option for each of the questions below.

	hardly ever	some of the time	often
How often do you feel that you lack companionship?			
How often do you feel left out?			
How often do you feel isolated from others?			
How often do you feel lonely?			

5.3 Thinking of the last two weeks, please let us know to what extent you have experienced any of the following.

	Not true at all				Very true
	1	2	3	4	5
I have experienced a lot of understanding and security from others					
I have a very close person whose help I can always count on					
If necessary, I can easily borrow something I need from neighbours or friends					
I have several people with whom I can do activities					
If I get sick, I have friends and family who will take care of important matters for me					
If I am feeling down, I have people I can go to without hesitation					

6. Use of the internet and digital devices

Here we ask if/how you have used the internet during the pandemic restrictions.

6.1 How would you rate your knowledge to use the Internet?

- ☐ Outstanding
- ☐ Good
- ☐ Fair
- ☐ Poor
- ☐ Bad
- ☐ Don’t know/can’t say

6.2 Do you own any of the following devices? (choose all that apply)

- ☐ Smartphone
- ☐ Tablet
- ☐ Laptop or desktop computer
- ☐ None of these

6.3 Can you access the internet from your home (e.g. broadband/data on phone)?

- ☐ Yes
- ☐ No

6.4 In general, have you used the internet during the pandemic restrictions to do some of your daily activities (e.g. buy groceries, pay bills, etc.)?

- ☐ Yes, a lot [complete question 6.5 and skip question 6.6]
- ☐ Yes, a little [complete both 6.5 and 6.6]
- ☐ No [go to question 6.6]

6.5 Since the pandemic restrictions began, have you been using the internet to do any of the following?

	Yes	No
To stay in touch with colleagues from work		
To stay in touch with friends/family		
To pay bills		
To buy groceries or order food		
To buy other things I needed, other than food.		
For information (e.g. follow the news) and/or entertainment (e.g. movies or music)		
To get support (e.g. GP, psychiatrist, mental health team, or voluntary organisation)		

6.6 (Skip if 6.4 was a yes) **Here are some reasons explaining why people might not go online. Please let us know which of these may apply to you** (choose all that apply).

I am just not interested	
I have no connection where I live or where I work	
I have no computer, tablet or smartphone available	
It's too difficult to use	
It's not useful	
Having and accessing the internet is too expensive	
I am worried about my privacy	
I worry about bad experiences with SPAM or viruses	
I don't have enough time	
There is nothing of interest online	
I do not yet know how to use the internet	
It is too time consuming	
It is not for people of my age	
I worry about the security of my data and information	
Other (please specify)	

6.7 Would you like to learn more about how to use the internet to do some of your daily activities?

☐ There might be things I don't know, and I would be interested in learning
☐ There might be things I don't know but I am **not** interested in learning
☐ I already know how to do the things I want.

7. Employment

Here we would like to ask you about your work and how this might have been affected by the pandemic restrictions.

7.1 How would you describe your employment just before the beginning of the pandemic restrictions? Choose all that apply.

- ☐ Employed full-time (30+ hours/week)
- ☐ Employed part-time (<30 hours/week)
- ☐ Self-employed
- ☐ Retired
- ☐ Looking after family or home
- ☐ Student
- ☐ Voluntary worker (paid or unpaid)
- ☐ Not employed but seeking work
- ☐ Not employed but not seeking work because of ill health
- ☐ Not employed but not seeking work for some other reason
- ☐ other

7.2 Since the pandemic restrictions began has your employment status changed in any of the following ways? Choose all that apply.

My employment status has not changed	
Became unemployed	
Reduction in hours	
Reduction in salary	
Benefits increased	
Benefits decreased	
Change in duties or responsibilities	
Became employed	
Increased hours	
Increased salary	
Furloughed or paid leave	
Other	

7.3 Compared to before the pandemic restrictions, how would you say you are doing financially right now?

- ☐ I am better off
- ☐ I am worse off
- ☐ I am about the same
- ☐ I don’t know
- ☐ I do not wish to answer

8. Any other comments

In this section you can tell us about anything else you think is important for you.

8.1 Is there anything else you would like to add?

☐ No, no further comments

☐ Yes (please type your comments below)

Thank you for taking part

Your participation was valuable to understand the impact of the pandemic restrictions on people's health and wellbeing.

9. Invitation to Qualitative Interview

Are you interested in taking part in an interview?

By completing this survey, you and the other people who have taken part have given us really useful information about people's health and wellbeing during the pandemic restrictions. However, we would like to talk to a smaller group of people (around 15 to 20) in more detail about the things that have helped or been especially difficult for them during the pandemic restrictions.

If you would be interested in taking part in a short telephone interview, please tick the box below. A researcher from our team will contact you by phone in the next few weeks to give you more information and arrange a time to talk to you. If you say 'yes' now but change your mind later, that's fine, just tell the researcher when they ring.

9.1 I am interested in talking to a researcher about my experiences during the pandemic restrictions:

☐ Yes

☐ No

10. Invitation to MELLOW study

Are you interested in taking part in a future study about mental health and wellbeing?

We are grateful for your help in this survey. We also plan to run another study in the future, which you might be interested in taking part. We are looking for a group of people to complete a series of surveys over five years, to understand how various aspects of life might influence health and wellbeing in the long term. Your answers will help influence the future and improve the care of people with severe mental illness.

If you are interested, please tick the box bellow. A researcher from our team will contact you by phone or post in the next couple of months to give you more information. If you say 'yes' now but change your mind later, that's fine, just tell us when we contact you again.

10.1 I am interested in taking part in these future surveys:

☐ Yes

☐ No
